# DNA barcoding reveals diverse growth kinetics of human breast tumour subclones in serially passaged xenografts

**DOI:** 10.1038/ncomms6871

**Published:** 2014-12-23

**Authors:** Long V. Nguyen, Claire L. Cox, Peter Eirew, David J. H. F. Knapp, Davide Pellacani, Nagarajan Kannan, Annaick Carles, Michelle Moksa, Sneha Balani, Sohrab Shah, Martin Hirst, Samuel Aparicio, Connie J. Eaves

**Affiliations:** 1 Terry Fox Laboratory, British Columbia Cancer Agency, 675 West 10th Avenue, Vancouver, British Columbia, Canada V5Z 1L3; 2 Department of Molecular Oncology, British Columbia Cancer Agency, 675 West 10th Avenue, Vancouver, British Columbia, Canada V5Z 1L3; 3 Centre for High-Throughput Biology, Department of Microbiology & Immunology, University of British Columbia, 2125 East Mall, Vancouver, British Columbia, Canada V6T 1Z4

## Abstract

Genomic and phenotypic analyses indicate extensive intra- as well as intertumoral heterogeneity in primary human malignant cell populations despite their clonal origin. Cellular DNA barcoding offers a powerful and unbiased alternative to track the number and size of multiple subclones within a single human tumour xenograft and their response to continued *in vivo* passaging. Using this approach we find clone-initiating cell frequencies that vary from ~1/10 to ~1/10,000 cells transplanted for two human breast cancer cell lines and breast cancer xenografts derived from three different patients. For the cell lines, these frequencies are negatively affected in transplants of more than 20,000 cells. Serial transplants reveal five clonal growth patterns (unchanging, expanding, diminishing, fluctuating or of delayed onset), whose predominance is highly variable both between and within original samples. This study thus demonstrates the high growth potential and diverse growth properties of xenografted human breast cancer cells.

## Abstract (web summary)

Cancer cells within the same tumour are heterogeneous in their tumorigenic potential, differentiation status and sensitivity to treatments. Here Nguyen *et al*. use a sensitive DNA barcoding method to characterize the diversity of clonal growth behaviour within human breast tumours.

## Graphical abstract

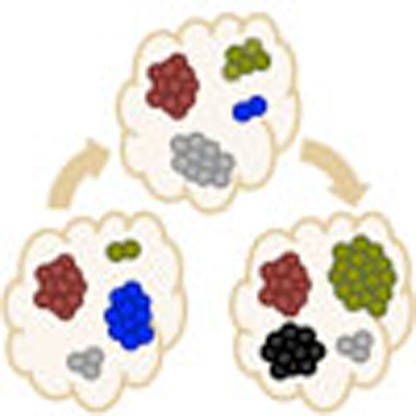

Malignant human breast populations are characterized by extensive heterogeneity in the genomic, phenotypic and biologic properties of the cells they comprise. The diversity of these elements is believed to contribute to the heterogeneity these cells display in their *in vivo* growth and responses to general as well as molecularly targeted treatments. One source of heterogeneity is believed to derive from an incomplete suppression of the process of differentiation that takes place in the normal adult mammary gland to regulate its composition and size^1^. This process includes the exclusive retention by rare subsets of cells of the property of self-renewal; that is, the ability to generate daughter cells that are not destined to die nor become unable to proliferate further by the activation of a terminal differentiation programme^2^. The presence of such cells in normal human breast epithelial cell populations has been demonstrated from experiments showing an association of *in vivo* regenerative activity with rare cells that display a distinct phenotype^3^,^4^, and a similar principle has been used to indicate a hierarchy of growth potential in breast cancers^5^. *In vitro* studies using non-adherent culture conditions have further strengthened this concept^6^. However, it is important to note that expression of many of the surface markers used to identify cells with tumour-initiating cell activity appears to be unstable^7^, and may also be variably associated with tumour-initiating potential^8^.

A majority of human breast tumours have acquired extensive genomic diversity by the time they become clinically evident, and whole-genome sequencing approaches have identified clonal genotypes that emerge or diminish^9^ presumably as a result of their response to a variety of selection pressures^10^. In contrast, limiting dilution assays (LDAs) are not constrained to detect clones based on their genotype. However, they are highly sensitive to the end point used to discriminate a ‘positive’ from a ‘negative’ result. In addition, LDAs are, by definition, unable to examine the concurrent growth behaviour of multiple subclones within a given tumour and that may have different kinetics of expansion. Hence, LDA experiments are unsuited to large-scale screens of agents that may influence tumour growth or recurrence.

Vector-marking strategies also enable transplanted clonal growth patterns to be investigated, either by identifying unique sites of randomly integrated vector DNA into the genome of each transduced cell, or by identifying unique vector DNA sequences (referred to as ‘barcodes’) integrated at single copy level into individual cells exposed to a highly diverse library of viral vectors^11^. Use of the first approach has shown biologically determined as well as chemotherapy-induced variations in the clonal growth dynamics of malignant human cells in xenografts initiated with different primary sources of tumour cells, as well as different established cancer cell lines^12^,^13^,^14^,^15^. Cellular barcoding offers an attractive alternative strategy to address these issues because of its potentially greater sensitivity of clone detection^16^,^17^,^18^,^19^, as recently illustrated in analyses of cells produced by normal human hematopoietic and mammary cells transplanted into immunodeficient mice^19^,^20^. Here, we demonstrate the highly complex clonal growth dynamics that barcoding reveals in serially passaged xenografts of widely used cell lines originally derived from established human mammary cancers and related complexity in barcoded transplants of cells more immediately derived from primary human breast cancers.

## Results

### Input cell dose affects clone formation in xenotransplants

**Figure 1:**
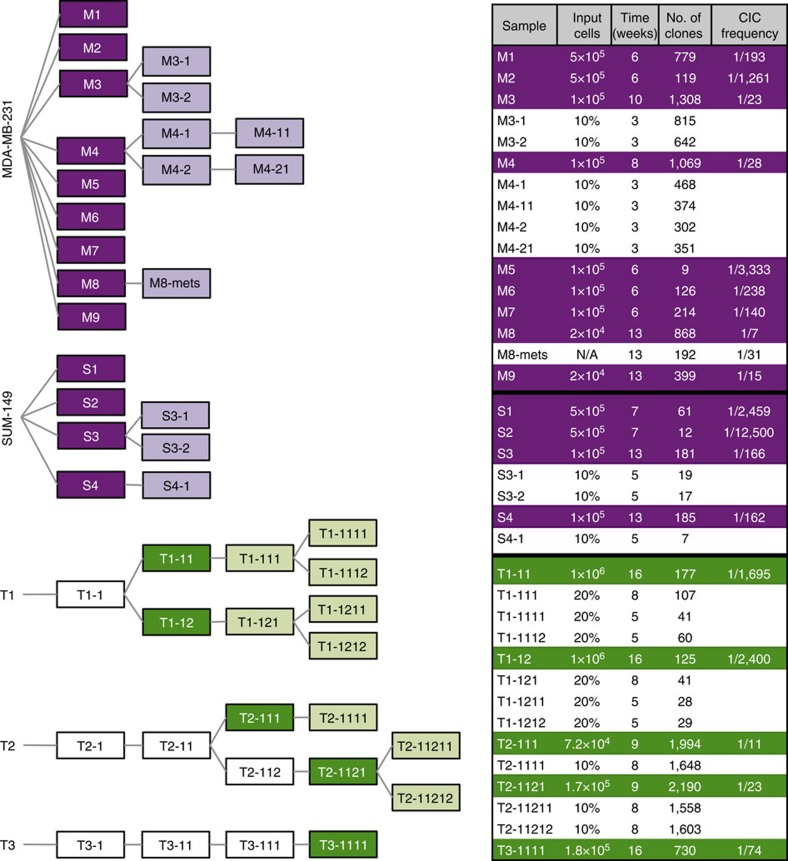
Experimental design. Serial passages of individual tumours derived from separate samples of barcoded MDA-MB-231 and SUM-149 cells (nine and four, respectively) are shown in purple, and for cells from patient-derived xenografts in green. White boxes indicate early passages of patient-derived xenografts, before barcoding cells for subsequent clonal analyses. The total number of cells (or fraction of the previous tumour) used to initiate each subsequent tumour, the time before removing the tumour for analysis, and the number and frequency of uniquely barcoded clones (expressed as a proportion of the estimated input number of barcoded cells) are shown on the right. CIC frequencies were calculated as the no. of clones divided by the total no. of barcoded cells transplanted based on the 30% transduction efficiency measured by FACS analysis of input cells.

As a first experimental model of human breast cancer growth *in vivo*, we injected separate groups of highly immunodeficient female *NOD/SCID/IL2rγ*^*−/−*^ (NSG) or *NOD/RAG1*^*−/−*^*IL2rγ*^*−/−*^ (NRG) mice subcutaneously (on the back) with cells from two widely used human breast cancer cell lines: MDA-MB-231 and SUM-149 cells. To measure the number and size of clones present in the tumours subsequently generated, we exposed the cells just before the transplant with a previously described lentiviral library containing thousands of different DNA barcodes under conditions designed to obtain a single unique barcode in each cell transduced^19^. A total of 13 mice were injected subcutaneously with 2 × 10^4^ to 50 × 10^4^ cells, of which 30–35% were shown to be transduced (GFP^+^) and hence could be assumed to be individually barcoded^11^,^19^. Palpable tumours were apparent in all mice within 7 to 13 weeks, depending on the number of cells transplanted (Fig. 1). Approximately 10% of each harvested tumour was then used to determine the number and frequency of barcodes present using massively parallel sequencing of barcode-containing amplicons and a series of spiked-in controls included in each test sample (see Methods for details). The controls allowed us to set a conservative threshold for clone detection at a fractional read value (FRV) equivalent to 300 cells, based on the finding that this FRV allowed clones containing more than 500 cells to be detected at 90% sensitivity (9 of 10 control samples detected, with an inferred detection efficiency of 100% for clones of more than 5,000 cells) and an associated specificity of 99.7% (only one false-positive clone among 390 examined in 10 control cell samples, Supplementary Fig. 1 and Supplementary Table 1).

**Figure 2:**
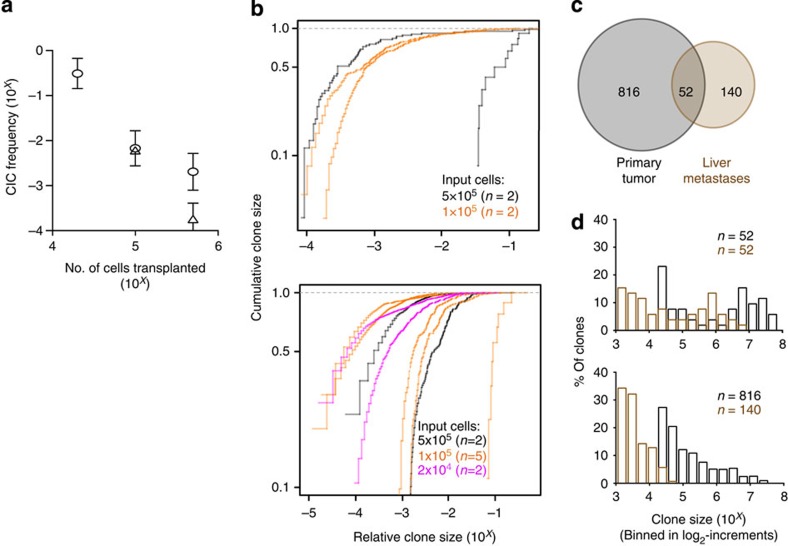
Variation in clone size in primary and metastatic tumour xenografts. (**a**) An inverse linear relationship is seen between the numbers of cells transplanted and the number of clones detected, for both cell lines tested (Δ SUM-149 cells; ○ MDA-MB-231 cells). Values shown are the geometric mean±s.e.m. of the frequency of CICs calculated for each of the tumours identified in Fig. 1. (**b**) Cumulative distributions of clone sizes in primary xenografts generated from different numbers of S1–S4 (upper panel) and M1–M9 cells (lower panel). (**c**) Overlap between clones present in the tumour (M8) that arose at the site of injection (black) and in simultaneously assessed liver metastases (brown). (**d**) Comparison of the distributions of the 52 overlapping clones identified in panel c (upper panel) and for the 816 and 140 clones detected simultaneously at the injection site (black) and liver (brown), respectively (lower panel). The *x*-axis represents the size of clones binned in log_2_-increments.

Using this FRV threshold to define the presence of a clone, we then quantified the number of clones present in the tumours obtained (Fig. 1). In mice transplanted with MDA-MB-231 cells (M1–M9), the total number of barcoded clones thus detected in each tumour varied from 9 to 1,308. From these numbers and the number of transduced MDA-MB-231 cells originally transplanted into each mouse, we calculated the frequency of barcoded cells that had generated a detectable clone. Surprisingly, the ‘clone-initiating cell’ (CIC) frequency values thus obtained were not constant, but decreased over more than two orders of magnitude (from 1 in 7 to 1 in 3,000 of the cells transplanted) as an *inverse* function of the input cell dose over the 25-fold range of cell doses tested (from 2 × 10^4^ to 50 × 10^4^ total cells per mouse, Fig. 2a). Similar results were obtained for the tumours generated from 10 × 10^4^ to 50 × 10^4^ SUM-149 cells (S1–S4). In this case, the corresponding number of detectable clones decreased 15-fold (from 185 to 12), resulting in detectable CIC numbers that decreased from 1 in 160 to 1 in 12,500 of the cells transplanted.

### Highly variant clonal growth of cell lines *in vivo*

The barcode data also allowed clone size measurements to be related to the source and dose of the cells transplanted. To accommodate the different sizes of tumours being compared, each clone size was normalized to the total size of the tumour in which it was present. Cumulative distribution plots of these normalized clone size values for each of the 13 tumours generated from the MDA-MB-231 and SUM-149 cells showed significant variation in the relative clone sizes produced both within and between tumours, but without a clear input cell dose effect (Fig. 2b). The largest range in clone size within a single tumour was observed in the M3 tumour (>10^4^–fold). Between tumours, even the largest clones spanned a similar range despite similar inputs.

Macroscopically obvious metastases were noted in the liver of a single mouse that had been injected with the lowest dose (2 × 10^4^) of MDA-MB-231 cells (tumour M8). Analysis of the number of unique barcodes detected in the bulk liver cells obtained from this mouse indicated the presence of 192 clones (Fig. 2c). Notably, the majority (140) of the 192 metastatic clones were not detected in the tumour that arose subcutaneously at the initial site of M8 cell injection, and were relatively small, ranging in size from 10^3^ to 4 × 10^4^ cells per clone (Fig. 2d). The other third of the metastatic clones arose from cells whose progeny were readily detected in the tumour that arose at the site of injection. They also reflected the same bimodal clone size distribution (with upper and lower modal clone size values at both sites differing by a factor of ~10), although, on average, those present in the liver were again smaller (Fig. 2d).

These results demonstrate the sensitivity of vector-mediated barcoding to detect and monitor a wide range of CIC activity that can be displayed by malignant human cells proliferating in tumour xenografts and derived metastases. They also show that, at least for the cell lines tested here, manifestation of CIC activity is highly negatively affected when the number of cells initially injected is in the range commonly used.

### Passaged cell lines exhibit changing clonal growth patterns

To monitor the stability of the diverse clonal growth activity exhibited by the MDA-MB-231 and SUM-149 cells that generated primary tumours, we transplanted 10% of the cells harvested from four of the primary tumours (M3, M4, S3 and S4) into secondary mice. In each case, palpable tumours were detected 3 to 5 weeks later, at which time these secondary tumours were harvested for analysis of their barcode content, except for M4, where 10% of the cells were used to generate tertiary tumours (Fig. 1). The barcode data obtained from these passaged tumours indicated that they contained consistently fewer clones than the primary tumours from which they had been derived, although in the matched pairs (derived from the same parental tumour), the reduction in content of barcoded cells was similar (approximately twofold for the passaged cells in the M3 and M4 experiments, and approximately sixfold for the passaged cells in the S3 experiment). However, both the frequency of all clones that were detectable at every passage and their individual prominence was highly variable.

**Figure 3:**
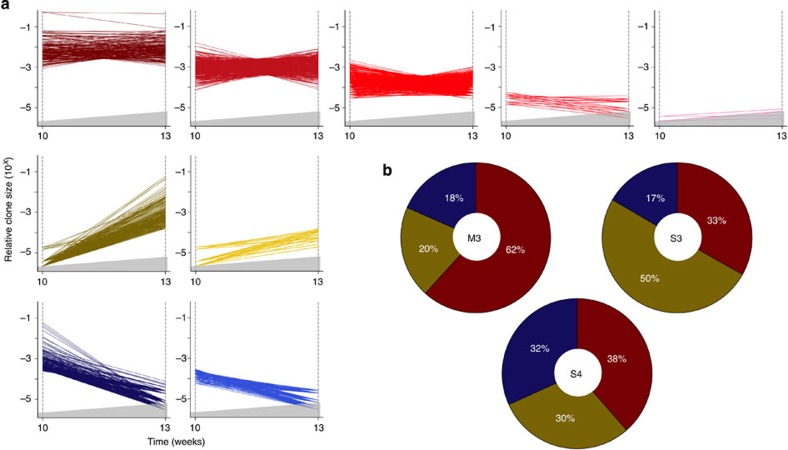
Diverse *in vivo* clonal growth patterns of human breast cancer cell lines. (**a**) Growth patterns of individual clones in primary and secondary derivative tumours generated from M3 cells. In each plot, a separate line portrays the growth activity of an individual clone in successive passages. Clones that remained relatively constant in size between passages are shown in shades of red, and those whose size increased or decreased are shown in shades of yellow and blue, respectively. The area in each plot shaded in grey represents the relative clone size below the threshold used for detecting barcoded clones. In cases where replicate tumours had different limits of detection, and are represented on the same plot, the higher limit is shown. (**b**) Relative proportions of the different clonal growth patterns at each passage from M3, S3 and S4 cells. Colours in each sector correspond to the colour-coded clonal patterns described in **a**.

To enable a more comprehensive comparison of the growth patterns exhibited by different clones in sequentially generated tumours, we used k-means clustering to group clones according to the measured change in size of each on sequential passaging (Supplementary Fig. 2). For the M3 experiment, this analysis identified three general patterns (Fig. 3a). The most prevalent of these (displayed by 62% of all M3 clones detected) was one where the relative clone size remained constant. The least prevalent pattern (displayed by 18% of all M3 clones detected) was one where the relative clone size decreased in the second passage. The remaining 20% of all M3 clones detected displayed a pattern where the relative clone size increased with passaging (Fig. 3b). Within each of these groups, additional subgroups were also resolved according to the relative size of each clone and the magnitude of its change in size (Fig. 3a).

**Figure 4:**
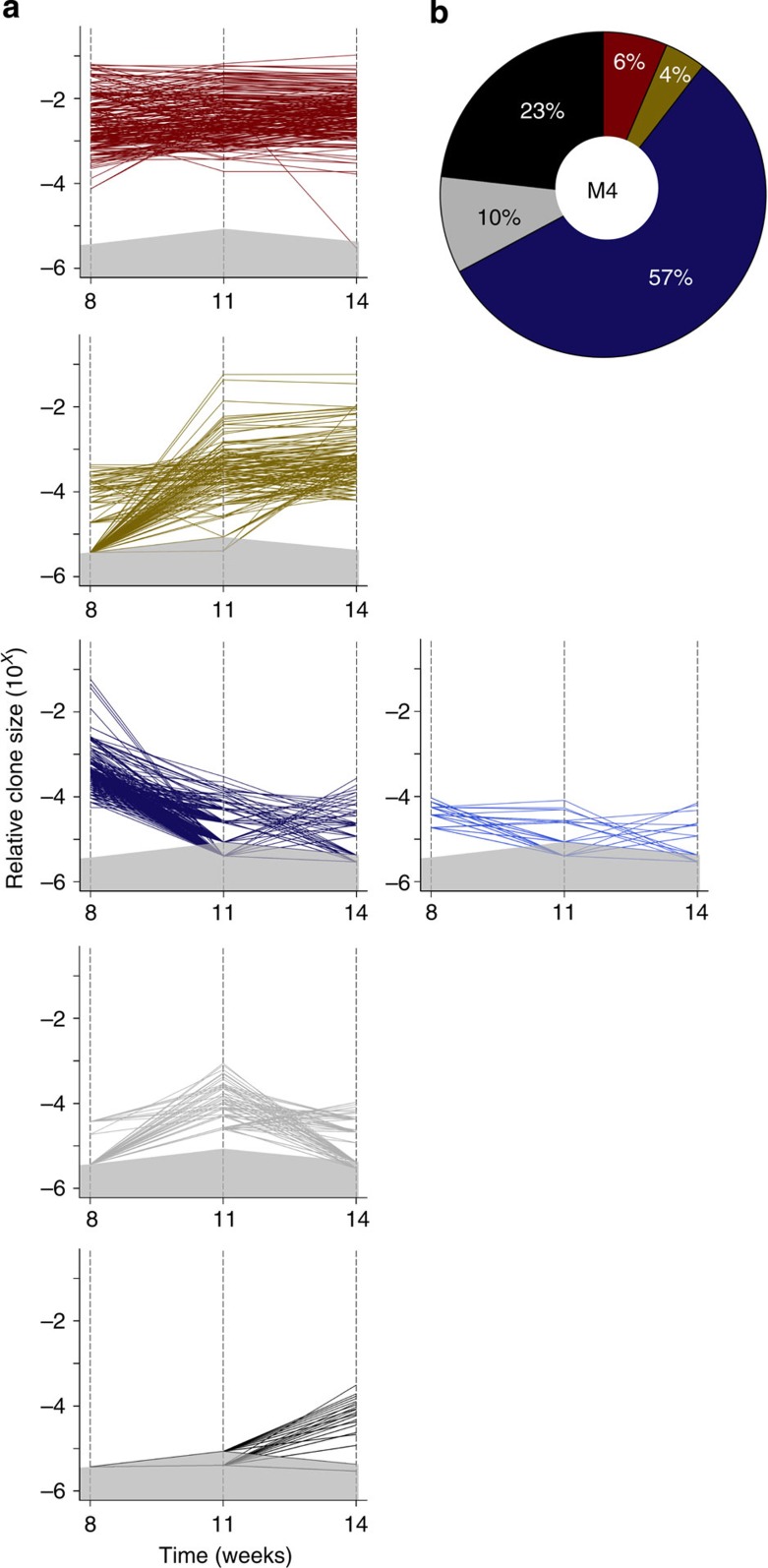
Delayed growth of M4 clones in serially transplanted mice. (**a**) Growth patterns of individual clones in primary, secondary and tertiary tumours derived from M4 cells. In each plot, a separate line portrays the growth activity of an individual clone in successive passages. Clones that remained relatively constant in size between passages are shown in shades of red, and those whose size increased or decreased are shown in shades of yellow and blue, respectively. Clones that first became detectable in secondary and tertiary tumours are shown in grey and black, respectively. The area in each plot shaded in grey represents the relative clone size below the threshold used for detecting barcoded clones. In cases where replicate tumours had different limits of detection, and are represented on the same plot, the higher limit is shown. (**b**) Relative proportions of the different M4 clonal growth patterns at each passage. Colours in each sector correspond to the same colour-coded clonal patterns described in **a**.

For the M4 experiment, the k-means cluster analysis identified the same three groups but with fewer subgroups (Fig. 4a) and the prevalence of their different growth patterns was reversed. Thus, the most prevalent pattern (displayed by 57% of all M4 clones detected) was for the clones that became smaller after the first transplant, and the least prevalent pattern was where clones remained constant or increased in size (6 and 4%, respectively, of all M4 clones detected, Fig. 4). Interestingly, in this experiment, k-means clustering identified two additional groups, both characterized by delayed clonal growth; that is, clones that were not detectable until the formation of secondary, or even tertiary tumours, and these comprised 10 and 23%, respectively, of all clones identified. Notably, many of the clones that became detectable in the secondary tumours, subsequently, decreased in tertiary tumours, indicative of a fluctuating growth pattern.

In the S3 and S4 experiments, seven and six clusters, respectively, were identified, and these also included clones whose sizes remained constant, expanded or diminished (Fig. 3b and Supplementary Fig. 3) and their proportions quite similar in both experiments: 33, 50 and 17% of all clones detected, respectively, in experiment S3, and 38, 30 and 32% of all clones detected in experiment S4.

These findings illustrate the unbiased and strong resolving power of DNA barcoding to reveal distinct patterns of clonal growth in a complex landscape of behaviour obtained in highly polyclonal xenografts of malignant human cell lines.

### Diverse *in vivo* dynamics of CICs from patients’ tumours

We then conducted a similar series of experiments with cells obtained from xenografts of three patients’ breast cancers (Fig. 1). One of these xenografts had been generated from a pleural effusion that developed in a patient with an advanced stage (originally ERα^+^) tumour. In this case, the cells to be barcoded were obtained from a primary xenograft (T1). Barcoding was also applied (separately) to cells from a secondary and a tertiary xenograft originally generated from another primary ER^*−*^PR^*−*^HER2^*+*^ tumour (T2), as well as to a tertiary xenograft of cells originally from a third, primary ER^−^PR^−^HER2^+^ tumour (T3)^10^. Aliquots of 0.7 × 10^5^ to 10 × 10^5^ cells from these xenografts were injected into NSG mice immediately post-transduction and the efficiency of transduction shown to be ~30%, based on the proportion of cells that were GFP^+^ in an aliquot kept in culture for another 2 days. Tumours derived from the barcoded cells became evident 9 to 16 weeks post-transplant. Each of these was then passaged another 2–3 times with paired replicates, as indicated in Fig. 1.

In the 10% sample removed from the cells harvested from the first of the tumours thus generated, we identified 6 × 10^6^ to 9.6 × 10^8^ barcoded cells and these were distributed among 125 to 2,190 different clones. CIC frequencies calculated from these clone numbers (divided by the number of barcoded cells injected) varied over a >100-fold range (from 1/11 to 1/2,400 cells transplanted) for the different tumour sources, although values were quite consistent between paired replicates. Interestingly, the total number of clones detected in each tumour decreased in later tumour passages, as had been observed for the tumours generated from the human breast cancer cell lines.

**Figure 5:**
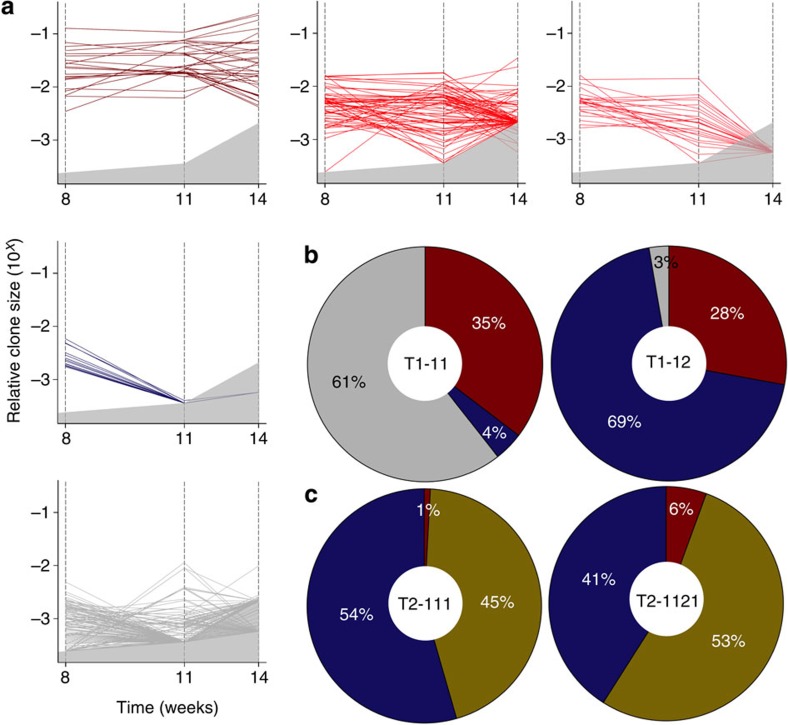
Diverse *in vivo* clonal growth patterns of patient-derived tumour xenografts. (**a**) Growth patterns of individual clones in primary, secondary and tertiary tumours generated from T1-11 cells. In each plot, a separate line portrays the growth activity of an individual clone in successive passages. Clones that remained relatively constant between passages are shown in shades of red, and those whose size decreased are shown in blue. Clones that first became detectable in secondary tumours or that fluctuated in size between passages are shown as grey. The area in each plot shaded in grey represents the relative clone size below the threshold used for detecting barcoded clones. In cases where replicate tumours had different limits of detection, and are represented on the same plot, the higher limit is shown. (**b**) Relative proportions of the different clonal growth patterns exhibited by T1-11 and T1-12 cells. Colours in each sector correspond to the colour-coded clonal patterns described in **a**. (**c**) Relative proportions of the different clonal growth patterns exhibited by T2-111 and T2-1121 cells. Colours in each sector correspond to the colour-coded clonal patterns described in **a**, except for clones that increased in size between passages that are shown in yellow.

We also used k-means clustering to determine the number and types of different clonal growth patterns obtained in these patient tumour-derived xenografts (Supplementary Fig. 4). Analysis of the data from the T1-11 and T1-12 tumours showed these shared only three of the patterns exhibited by the serially passaged cell lines; that is, no change, a decreasing, or a fluctuating change in clone size, and did not include clones that increased in size or showed a delayed onset of growth (Fig. 5a and Supplementary Fig. 5). There was also a marked variation in the relative prevalence of each pattern in T1-derived clones. For example, clones that fluctuated in size were highly prevalent in T1-11 (61% of all clones in that series), whereas, in T1-12, clones that diminished in size were the most prevalent (69%, Fig. 5b). Moreover, approximately one-third of the T1-11 clones that displayed a fluctuating behaviour were present at a detectable level in the first tumours studied, but then diminished in the next passage and reappeared in a subsequent passage. The other fluctuating T1-11 clones exhibited an opposite pattern in which they were not detected initially but became detectable in the next passage and then subsequently decreased, or first became detectable in the third passage. In contrast, for T2, a continuous increase or decrease in clone size was the most prevalent patterns seen (displayed by 45 and 54%, respectively, of T2-111 clones, and 53 and 41%, respectively, of T2-1121 clones, Fig. 5b and Supplementary Fig. 6). Thus, the presence of fluctuating clones and the high variability in prevalence of different growth patterns between replicates for T1 more closely resembles the behaviour of MDA-MB-231 cells, whereas the presence of only three main growth patterns and their relative reproducibility between replicates for T2 resembles the behaviour of SUM-149 cells.

These results reveal the extensive heterogeneity in the clonal growth dynamics seen both within and between tumour xenografts derived from different minimally xenografted human breast cancer cells from patients, as well as some similarities to the two human breast tumour cell lines studied.

### Asymmetry of growth activity in paired replicate tumours

**Figure 6:**
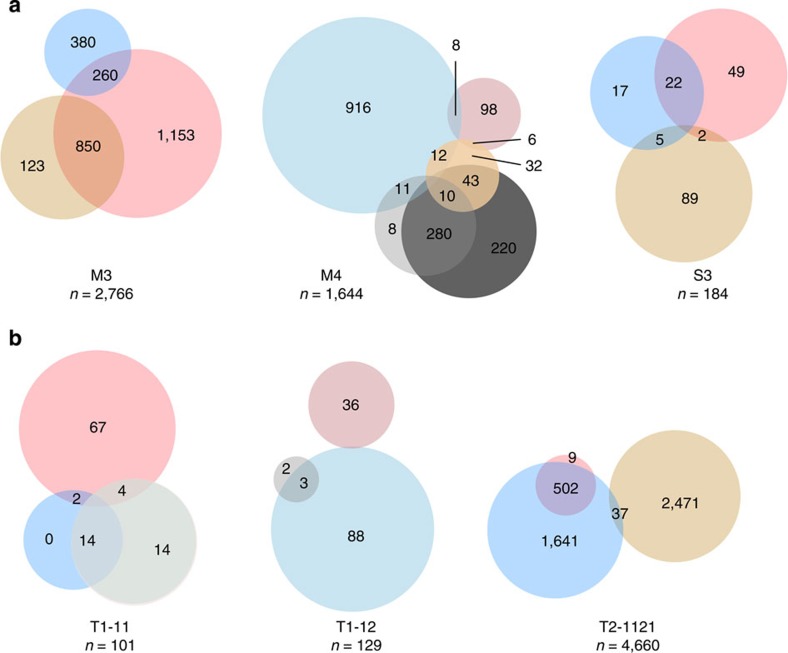
Replicate xenografts include both symmetric and asymmetric clonal growth patterns. (**a**) Venn diagrams showing the proportion of clones that demonstrated symmetrical (non-overlapping parts of the circle), and asymmetrical growth patterns (overlap between two circles) in replicate tumours derived from parental M3, M4 and S3 tumours. Different colours are used to identify each of the five clonal growth patterns detected as follows: constant (red), increasing (yellow), diminishing (blue), fluctuating (first appearing in secondary tumours, grey) and delayed (first appearing in tertiary tumours, black), and the size of each circle reflects the relative abundance of clones displaying the growth pattern it represents. The numbers shown are the absolute numbers of clones whose replicate derivatives displayed symmetrical or asymmetrical growth patterns. (**b**) Venn diagrams showing the proportion of clones that demonstrated symmetrical (non-overlapping parts of the circle) and asymmetrical growth patterns (overlap between 2 circles) in replicate tumours derived from parental T1 and T2 tumours, using the same colour coding and illustrative principles as in panel **a**.

To evaluate the extent to which these varying clonal growth dynamics might reflect random events, we compared the patterns obtained in pairs of secondary tumours generated from the same primary tumour (that is, from M3, M4, S3, T1-11, T1-12 and T2-1121). These paired comparisons showed that the majority of replicate clones (60–98%) were symmetrical in their subsequent growth patterns (Fig. 6). Experiments M3 and M4 demonstrated the most asymmetry in growth activity (33 to 40% of replicate clones showed different growth trajectories, Fig. 6a), and this usually occurred when the progeny of a clone increased or decreased in size in one daughter tumour only. Interestingly, the clones that most frequently displayed a symmetrical behavior in paired derivative tumours were those whose initial growth was delayed (~90% of the clones that were first detected in secondary tumours showed a replicate clone first detected in tertiary tumours).

In the patient-derived xenografts from tumour T1, 80 to 98% of replicate clones showed symmetry of growth activity, and the majority of asymmetry was displayed by clones whose initial size subsequently decreased in one derivative tumour and fluctuated in the other (44 to 60% of all fluctuating clones, showed a decline in the replicate tumour, Fig. 6b). In the xenografts derived from T2, 98% of the clones that maintained a relatively constant size between passages in one replicate showed a declining size in the other.

These analyses demonstrate a strong similarity in the growth behaviour of many clones as assessed in their paired derivatives, although examples of all possible combinations were also detected (Fig. 6).

## Discussion

Here we describe the feasibility, reproducibility and high resolving power of DNA barcoding as a method for tracking the clonal growth patterns of both established breast cancer cell lines and patient-derived breast cancer cells in tumours produced in immunodeficient mice. Our experiments produced several surprising outcomes. First was the overall qualitative similarity between the results obtained with cell lines and primary patient-derived tumour cells. Second was the decreasing number of clones detected in tumours initiated with increasing numbers of cells from established lines over a range of transplant doses that are widely used in other types of experiments. This finding suggests that clonal growth *in vivo*, even by very aggressive established cell lines transplanted into a highly permissive environment, is not autonomously determined. Rather it is subject to strong, as yet unexplored, negative effects from co-transplanted cells with strong tumorigenic potential. This finding poses significant difficulties in interpreting effects of any perturbation of tumorigenic capacity using such transplant models. Thus, at the lowest transplant doses tested (~2 × 10^4^ cells), the frequencies of cells that generated a clone of at least 300 cells in the tumours in which they were found ranged from ~5 to ~15%, that is, much higher than those frequently reported^21^, particularly for primary breast cancer xenografts^5^. Such high CIC frequencies argue against the concept of extreme developmental hierarchies in clinically detectable malignant human mammary cell populations, as well as those that have adapted to grow *in vitro*. The third unexpected finding was the large diversity of growth patterns observed between clones derived from all sources tested that became exaggerated with serial passage. Although many examples of replicate behaviour were also documented, substantial variation in longitudinal kinetics was also seen.

Particularly noticeable for tumours generated from MDA-MB-231 cells and recapitulated in xenografts derived from cells from patient sample T1 was the initial transient growth of many clones that were replaced in subsequent tumour passages by clones that had not been previously evident. This fluctuating clonal growth behaviour is supportive of the concept that CIC activity may be variably gained or activated and then later lost or transiently arrested due to both unstable intrinsic properties of the cells and changing environmental conditions. Such a concept has been well supported for human melanoma and certain experimental models of breast cancer, but not previously indicated for patients’ breast cancers^22^,^23^. However, it does provide an attractive explanation for the ability of malignant clones to become dormant *in vivo* over long periods of time. In contrast, instability in malignant growth properties could contribute in an important way to the biologic heterogeneity of a tumour in addition to mechanisms determined by specific genomic changes.

In contrast, in tumours generated from SUM-149 cells, initially dormant clones were relatively rare. Instead, the most prevalent clones were those that grew continuously in size with serial passaging. We have previously observed that a significant proportion of clones generated from normal mouse or human mammary cells as well as from human cord blood haematopoietic cells was similarly activated to display different growth potentials in secondary recipient mice^19^,^20^, a phenomenon found here to apply also to tumorigenic cell populations, even those present in established cell lines.

Analysis of tumour growth at single CIC resolution using a method that allows, but is not limited to, the detection of very large numbers of CIC activity in the same tumour adds an important new dimension to studies of the changing clonal landscape of patient xenografts. Assessment of how this landscape is shaped over time has not been feasible with previous methods. Our results demonstrate a much greater and continuing diversity in the growth activities of the individual clones that contribute to the combined exponential growth of tumours, and are shared between relatively ‘homogeneous’ cell lines^24^,^25^ and genomically highly heterogeneous patient xenografts^10^. The ready applicability of cellular barcoding to large-scale analyses of tumour growth and responses to treatment *in vivo* should thus add importantly to a growing armamentarium of tools to investigate underlying mechanisms and develop improved therapeutic approaches.

## Methods

### Cells

MDA-MB-231 and SUM149 cells were obtained from Dr Sandra Dunn (Child and Family Research Institute, British Columbia, Canada), and maintained mycoplasma-free as cultures of adherent cells in DMEM/F12 (STEMCELL Technologies) with 5% fetal bovine serum (FBS, STEMCELL Technologies) and DMEM/F12 with 5%FBS, 5 μg ml^−1^ insulin, and 1 μg ml^−1^ hydrocortisone. Aliquots were then cryopreserved in DMEM/F12 with 50% FBS and 6% DMSO. Samples of patients’ breast tumours from surgical resections or pleural effusions were obtained with informed consent according to procedures approved by the Research Ethics Board of the University of British Columbia. Tumour samples were mechanically dissociated by first mincing with scalpels and then with a Stomacher (Seward) in DMEM/F12 media with 5% FBS. The resultant cell suspension was immediately injected into 5- to 10-week-old female NSG or NRG mice in an inoculum containing 1:1 DMEM/F12+5%FBS and matrigel (BD Biosciences). Tumours arising in mice were similarly mechanically dissociated, then either passaged again into mice or cryopreserved at −156 °C in DMEM/F12 containing 50% FBS and 6% dimethylsulfoxide (DMSO). Pleural effusion samples were first centrifuged on ficoll-hypaque and the low-density (<1.077 g ml^−1^) cells collected by centrifugation on ficoll-hypaque and cryopreserved as mentioned above. Samples T1, T2, and T3 correspond to samples previously published by Eirew *et al*.,^10^ SA429, SA500, and SA532, respectively. All samples were obtained with informed consent and handled according to guidelines approved by the Research Ethics Board of the University of British Columbia.

### Lentiviral transduction

MDA-MB-231 cells were thawed, pre-cultured for 2–3 days and then washed in cold Hank’s Balanced Salt Solution (STEMCELL Technologies) supplemented with 2% FBS (HF), suspended in DMEM/F12+5%FBS at a concentration of 1 to 2 × 10^6^ cells per 100 μl and transferred to non-tissue culture 96-well plates. Concentrated supernatant containing barcoded lentiviral particles (10^9^ infectious units per ml)^19^ was then added at a final concentration of 1:200 and the cells incubated for 4 h at 37 °C. The cells were then washed in cold HF and pre-cultured for 2–3 days in DMEM/F12+5%FBS before the cells were harvested and transplanted. Thawed SUM149 cells were similarly pre-cultured and transduced, but in DMEM/F12+5%FBS supplemented with 5 μg ml^−1^ insulin and 1 μg ml^−1^ hydrocortisone. Cryopreserved samples from patients’ tumours were thawed and transduced using the same protocol as for the MDA-MB-231 cells. For the experiments with cell lines, two separate transduction reactions were performed for each, and fractions of the cell suspension prepared for different transplant doses (2 × 10^4^, 10 × 10^4^ and 50 × 10^4^ cells per injection). A small fraction for each cell line was re-plated and cultured to analyse for the percentage of cells expressing GFP 48 h later.

### Xenotransplantation of patient-derived xenografts and cell lines

After lentiviral transduction, an inoculum containing the transduced cells in 1:1 DMEM/F12+5%FBS and matrigel (BD matrigel) was prepared and injected subcutaneously in a 100 to 150 μl volume under the dorsal skin of young (8 to 12 week old) adult female NSG or NRG mice that were bred and maintained and followed under SPF conditions in the Animal Resource Centre in the British Columbia Cancer Research Centre in accordance with protocols approved by the University of British Columbia Animal Care Committee. Two to five injections were performed for each cell line at each cell dose tested to detect significant changes in measures of clone size and frequency, if apparent. Mice were not randomized, and investigators were not blinded to allocation during experiments, but were later blinded during barcode analysis. After tumours developed to a palpable size, the mice were killed and the tumours removed and dissociated. Ten percent of each tumour cell suspension was set aside for barcode sequencing and analysis, 10–20% was passaged into secondary and/or tertiary mice (Fig. 1) and the remainder cryopreserved in the same cryopreservation media used for the cell lines. FACS analysis of a subset of the barcoded tumours in primary mice revealed that, for the tumours derived from cell lines and patient samples, the proportion of GFP^+^ (barcoded) cells was similar (30–40% GFP^+^ cells) or slightly higher than that measured in a fraction of the cells maintained *in vitro* following lentiviral transduction and measured 2–3 days later.

### Sequencing and analysis of cellular barcodes

Genomic DNA was extracted from tumour cell samples to each of which a group of defined numbers of spiked-in control cells, each containing known barcodes (and spanning a range of 250 to 10^6^ cells each) using a PrepGEM DNA extraction kit (ZyGEM), and transferred to a 96-well plate in which the researchers were blinded to their identity, though there was no randomization in the order of the samples. These samples were then treated identically, and the DNA was sequenced as previously described^19^. In brief, we introduced a separate fault-tolerant sequenced-based index to uniquely identify experimental groups using a plate-based library construction protocol. Barcode amplicons were generated in a 35-cycle PCR reaction using sequence-specific primers with adaptors compatible with Illumina PE1 and PE2 primers (Illumina). Then, in a second 8–15 cycle PCR reaction, they were pooled at equimolar ratios and loaded into a single flow cell for paired-end sequencing on an Illumina MiSeq platform using a custom index sequencing primer for the second read^26^. To improve cluster recognition, a control phiX library was spiked into the amplicon libraries before sequencing (7% by mole). Primer sequences were used as previously described^19^. All multiplexed samples with at least 20,000 sequence reads were included, based on the average read coverage of all the samples tested.

### Barcode data processing and analysis

Retrieval of barcode sequences was performed using custom scripts, designed to retrieve only barcodes with a minimum base quality of 20 that matched the constant regions of the 27 nucleotide barcode sequence in both the forward and reverse directions. Barcodes belonging to each sample were then combined if they were within three mismatches. In the case that two or more barcodes (with three or fewer nucleotide mismatches) were combined, the most abundant barcode was taken to represent the group, and the sum of the reads corresponding to the barcodes combined was used. The sum of the read counts corresponding to the unique barcodes for each sample was then calculated.

### Calculation of fractional read values

FRVs were calculated as the no. of barcode reads divided by the sum of barcode reads obtained in the corresponding spiked in controls containing 250, 500, 5 × 10^3^ and 5 × 10^4^ cells that were included in every test sample analysed.

The denominator of the FRV varied slightly between samples, and thus acted to normalize the relative ‘size’ of each experimental clone within and between samples. The relationship with the best-fit was plotted for the FRV of the spiked-in controls and the known number of input cells corresponding to each. In this case, the relationship was:


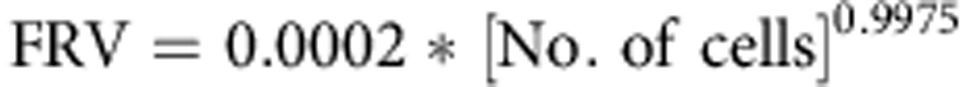


### Determination of the threshold used and consequent sensitivity and specificity of clone detection

A threshold was set at an FRV=0.059, equivalent to ~300 cells (on average for each of the samples sequenced) to maximize the sensitivity of clone detection but minimize the number of false barcodes not excluded by the threshold. To calculate the sensitivity and specificity of clone detection, 10 sets of samples that contained only spiked-in controls were used, each with a known barcode sequence, and added at known numbers of cells. Using these 10 sets of control-only samples, the FRV was calculated, and then any barcodes with an FRV<0.059 were excluded from the data set. This resulted in a sensitivity of 90% of clones containing 500 cells and 100% for clones containing 5,000 cells. The corresponding specificity was 99.7% because only one false-positive clone was detected among 390 others, in all 10 sets of control-only samples (meaning nine of these control-only samples did not have any false-positive clones).

### s.d. of the FRV for controls

The s.d. of the FRV for more than 5,000 control cells was ~0.06, but increased to ~0.3 for clones of less than 500 cells, reflecting the decreasing accuracy of barcode data to estimate the size of smaller clones. Thus, sampling error is higher for smaller clones.

### Calculation of absolute clone sizes

The FRV was then calculated for the clones in all the test samples, so that their relative sizes could be compared between samples, and the same FRV of 0.059 used as the threshold to eliminate any potential false positive barcodes. The absolute clone size (in cell numbers) for each clone was then calculated using the linear relationship between FRV and input numbers of cells:


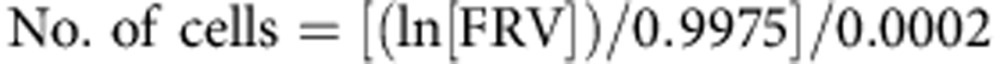


### Relative clone sizes

To compare the size of clones between passages in a given experiment, relative clone sizes were calculated as the no. of cells divided by the sum of the no. of cells for all clones in the tumour.

### K-means analysis

Clones were clustered based on their size in different passages using k-means analysis in R (version 3.1.1) with 100 re-starts and 100 iterations per run. The values for k were selected by inflection point analysis of the explained variability curve. This curve was estimated by running k-means clustering with varying k values (1–10 for clones with only two passages, 1–20 for clones with three passages) and calculating the explained variability (between-cluster sum of squares/total sum of squares) at each k value. A smoothened spline was then fit to this curve and the nearest k to the second positive inflection point was selected as this allowed automated and consistent selection of a k value at or near the point of diminishing returns.

## Author contributions

L.V.N. and C.J.E. conceptualized this project and wrote the manuscript. L.V.N., C.L.C., P.E., D.P. and S.B. performed the experiments. A.C. performed the computational and bioinformatics analysis of the sequencing data. M.M. and M.H. managed the deep sequencing of the libraries. L.V.N., P.E., D.J.H.F.K., D.P., N.K., S.S., M.H., S.A. and C.J.E. analysed and interpreted the data.

## Additional information

**How to cite this article:** Nguyen, L. V. *et al*. DNA barcoding reveals diverse growth kinetics of human breast tumour subclones in serially passaged xenografts. *Nat. Commun.* 5:5871 doi: 10.1038/ncomms6871 (2014).

## Supplementary Material

Supplementary Information Supplementary Figures 1-6 and Supplementary Table 1.
