# Supplementary material for: DNA barcoding reveals diverse growth kinetics of human breast tumour subclones in serially passaged xenografts
Source: Nat Commun. 2014 Dec 23;5:5871. doi: 10.1038/ncomms6871 (PMC4284657; doi:10.1038/ncomms6871)
Supplement: Supplementary Information — Supplementary Figures 1-6 and Supplementary Table 1. [file ncomms6871-s1.pdf]

SUPPLEMENTARY INFORMATION

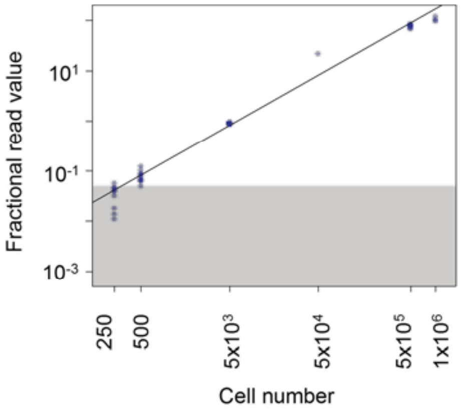

Figure S1. Detection threshold of barcoded clones
Demonstration of the linear (log-log) relationship found between the number of cells transplanted and the fractional read value (FRV) for each spiked in control used in the present experiments (each represented by a transparent blue point). The threshold (upper level of gray area) corresponds to a FRV of 300 cells. Details of the calculation are provided in the Methods of the main text. The proportions of clones detected using this threshold in 10 sets of controls that contained varying known numbers of cells previously shown to contain a single barcode are shown below in Table S1.

Table S1**. Sensitivity of detection of spiked in controls**

**No. of cells/clone**	**No. of clones detected above threshold/No. of clones tested (%)**
$1x10^6$	3/3(100%)
$5.5x10^5$	6/6(100%)
$5x10^4$	1/1 (100%
$5x10^3$	10/10(100%)
500	9/10(90%)
250	1/10(10%)

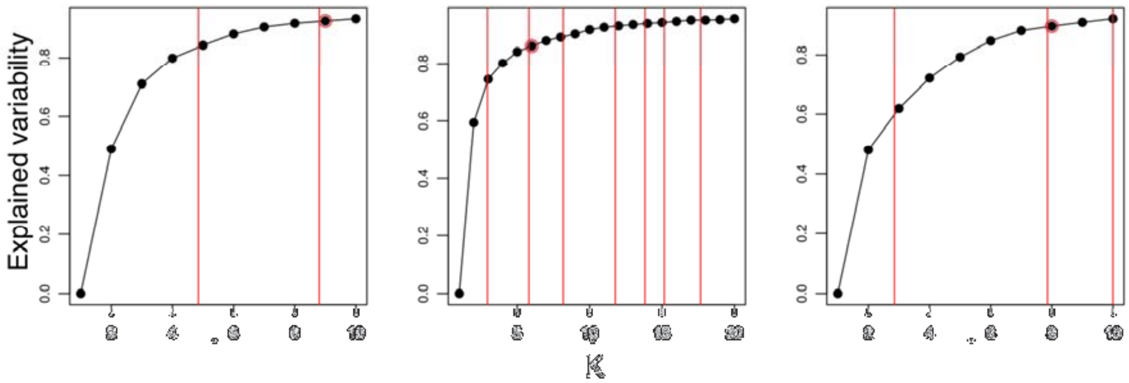

Figure S2. K value selection from groups of growth patterns displayed by MDA-MB-231 (M) and SUM-149 (S) CICs in tumour xenografts

Shown is the explained variance versus K-curves for changes in clone sizes in the experiments with M3 cells (left), M4 cells (middle), and S3 and S4 cells (combined, right). Positive inflection points are indicated with a vertical red line, and the K values selected (circled in red), correspond to the second positive inflection point in each experiment.

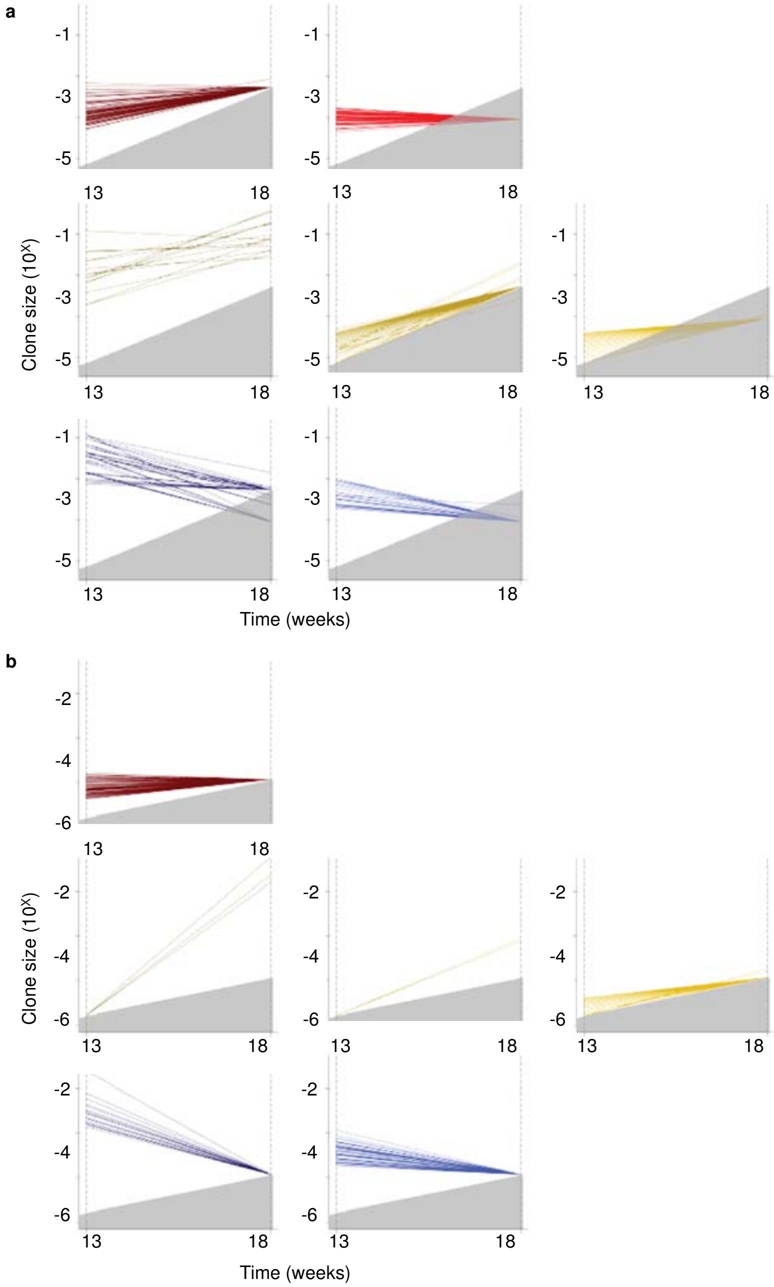

Figure S3. Clonal growth patterns displayed in serially passaged tumours generated from S3 and S4 cells as defined by K-means clustering

Clonal growth patterns identified by k-means clustering for S3 and S4 cells (a and b, respectively). Patterns are colour-coded according to their similarity to the growth patterns identified in Fig. 3 (i.e., patterns in which the clone size remained unchanged, or increased, or decreased are colour-coded in shades of red, yellow, and blue, respectively). The area in each plot shaded in gray represents the relative clone size below the threshold used for detecting barcoded clones. In cases where replicate tumours had different limits of detection, and are represented on the same plot, the higher limit is shown.

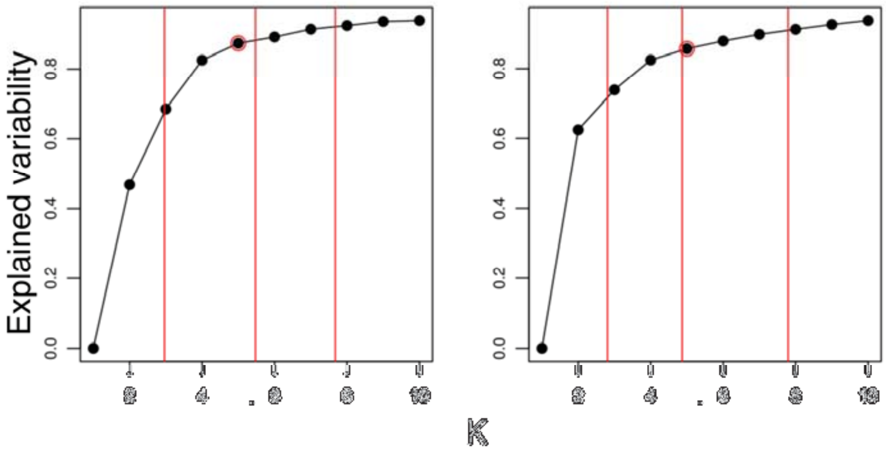

Figure S4. K value selection from groups of growth patterns displayed by breast cancer patient-derived malignant CICs in subsequent xenografts

Shown is the explained variance versus K-curves for changes in clone size in the experiments with T2-111 and T2-1121 cells (combined, left), and T1-11 and T1-12 cells (combined, right). Positive inflection points are indicated with a vertical red line, and the K values selected (circled in red) correspond to the second positive inflection point in each experiment.

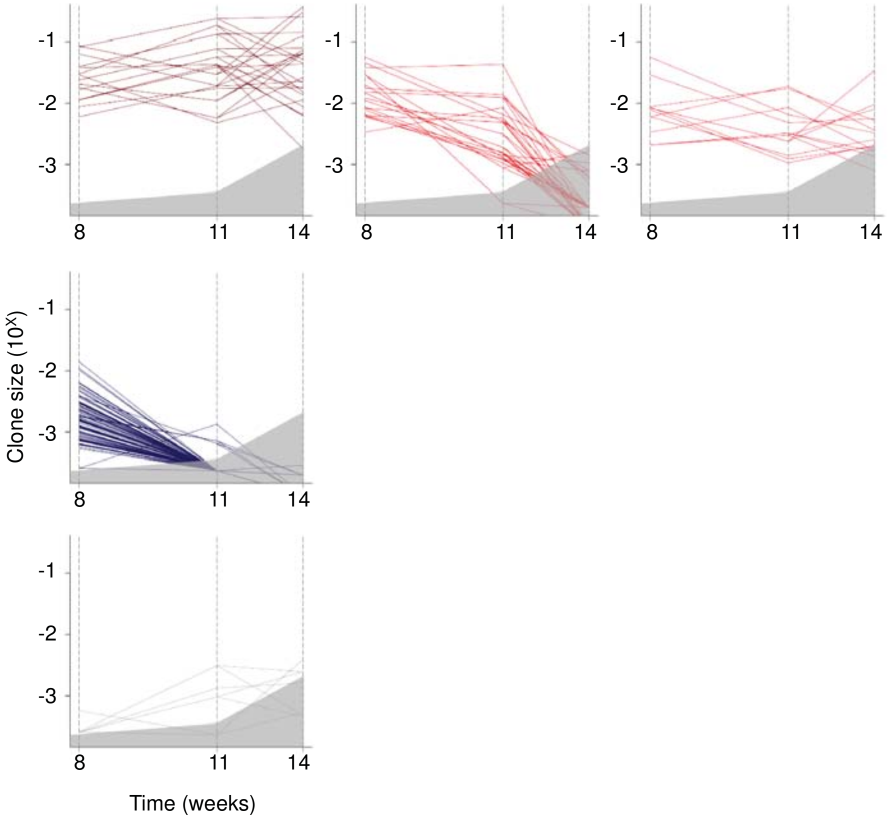

Figure S5. Clonal growth patterns displayed in serially passaged tumours generated from T1-12 cells as defined by K-means clustering

Clonal growth patterns identified by k-means clustering for T1-12 cells. Patterns are colour coded according to their similarity to the growth patterns identified in Fig. 5 (i.e., patterns in which the clone size remained unchanged, decreased or were first detected in secondary mice are colour-coded in shades of red, blue and gray, respectively). The area in each plot shaded in gray represents the relative clone size below the threshold used for detecting barcoded clones. In cases where replicate tumours had different limits of detection, and are represented on the same plot, the higher limit is shown.

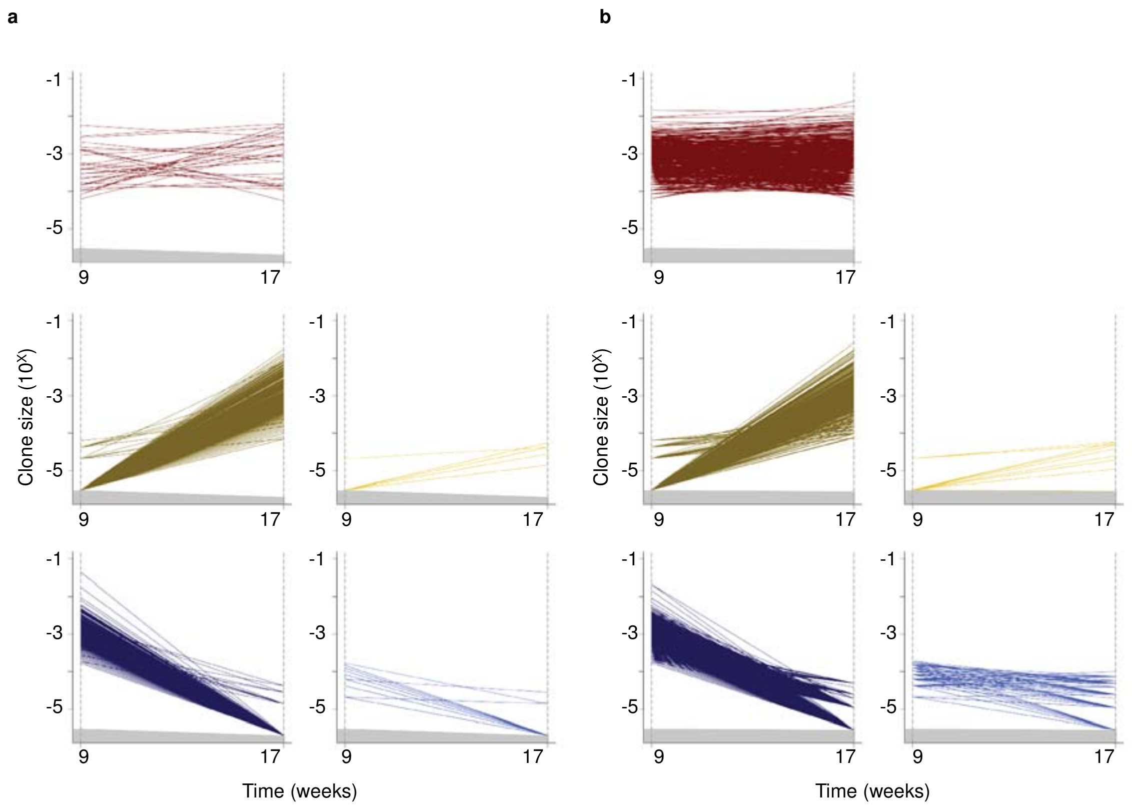

Figure S6. Clonal growth patterns displayed in serially passaged tumours generated from T2-111 and T2-1121 cells as defined by K-means clustering

Clonal growth patterns identified by k-means clustering for T2-111 and T2-1121 cells (a and b, respectively). Patterns are colour coded according to their similarity to the growth patterns identified in Fig. 3 (i.e., patterns in which the clone size remained unchanged, or increased, or decreased are colour-coded in shades of red, yellow, and blue, respectively). The area in each plot shaded in gray represents the relative clone size below the threshold used for detecting barcoded clones. In cases where replicate tumours had different limits of detection, and are represented on the same plot, the higher limit is shown.
